# Supplementary material for: Translation Inhibition by Rocaglamide A Enhances Susceptibility of Yeasts to Caspofungin
Source: bioRxiv. 2025 Sep 22:2025.09.21.677667. Preprint. [Version 1] doi: 10.1101/2025.09.21.677667 (PMC12485697; doi:10.1101/2025.09.21.677667)
Supplement: Supplement 1 [file NIHPP2025.09.21.677667v1-supplement-1.pdf]

bioRxiv preprint doi: https://doi.org/10.1101/2025.09.21.677667; this version posted September 22, 2025. The copyright holder for this preprint (which was not certified by peer review) is the author/funder, who has granted bioRxiv a license to display the preprint in perpetuity. It is made available under aCC-BY 4.0 International license.

Figure S1

RK-33

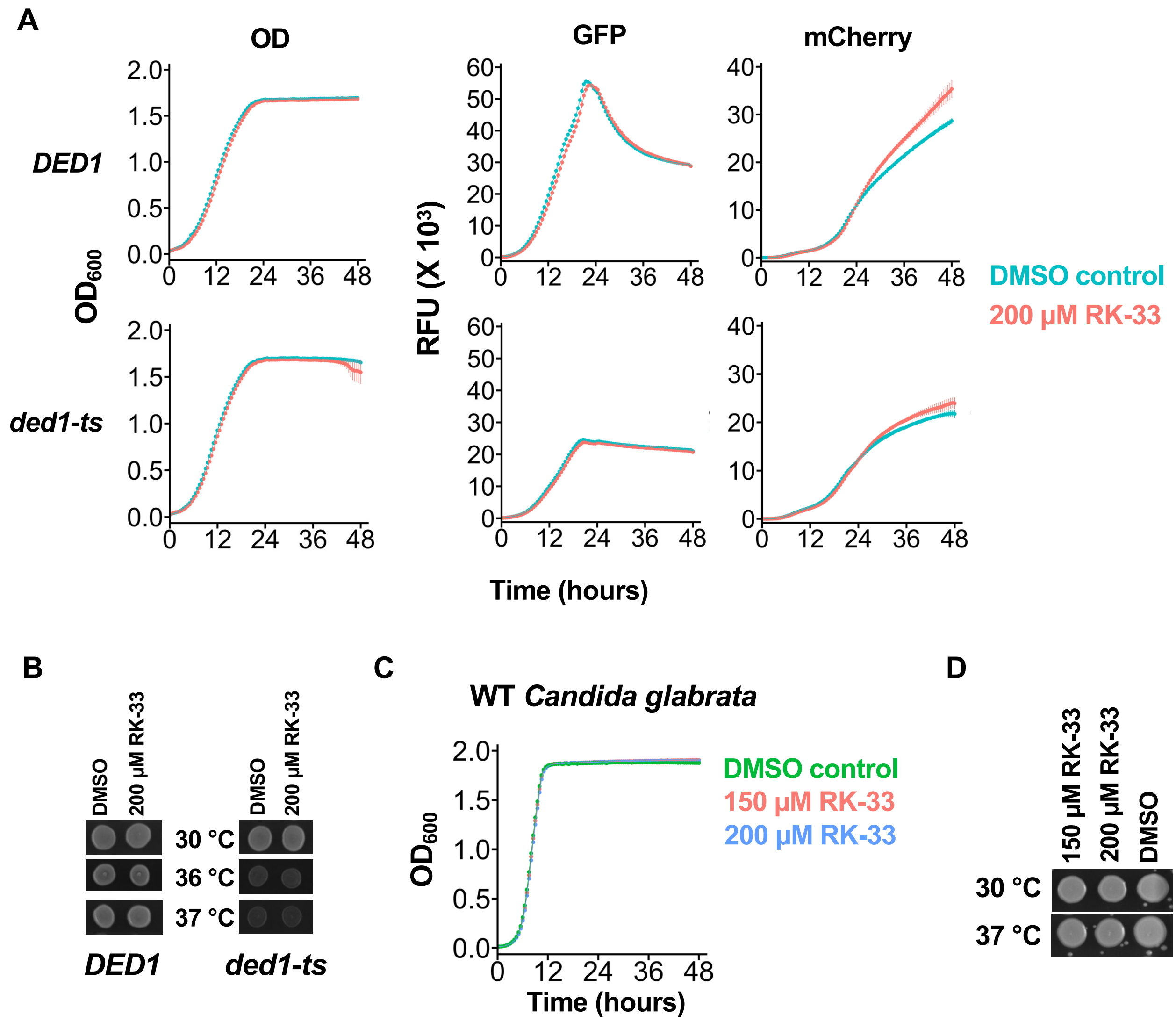

Figure S1. RK-33 was ineffective at inhibiting *S. cerevisiae* and *C. glabrata.* A. 48-hour growth curves of *DED1* (YSW223) and *ded1-ts* (YSW222) strains treated with 1% DMSO (no drug) or 200 μM RK-33 at 30°C. OD_{600}, GFP, and mCherry signals were measured simultaneously to assess growth and reporter translation. Data represents the mean ± standard deviation from 3 biological replicates **B.** Recovery assay following DMSO and RK-33 treatment. *DED1* and *ded1-ts* cells (YSW223 and YSW222) were spotted on YPD plates and incubated for one day at the indicated temperatures to evaluate survival. **C.** 48-hour growth curves of WT *C. glabrata* treated with 0, 150 μM or 200 μM RK-33. OD_{600} was measured to monitor growth. Data represents the mean± standard deviation from 4 biological replicates. **D.** Recovery assay following compound exposure: WT *C. glabrata* cells were spotted on YPD plates and incubated at indicated temperatures for one day.
